# Development of a risk score for colorectal cancer in Chinese males: A prospective cohort study

**DOI:** 10.1002/cam4.2734

**Published:** 2019-11-26

**Authors:** Lanwei Guo, Hongda Chen, Gang Wang, Zhangyan Lyu, Xiaoshuang Feng, Luopei Wei, Xin Li, Yan Wen, Ming Lu, Yuheng Chen, Jufang Shi, Jiansong Ren, Chunqing Lin, Xinyang Yu, Shuohua Chen, Shouling Wu, Ni Li, Min Dai, Jie He

**Affiliations:** ^1^ Office for Cancer Screening National Cancer Center/National Clinical Research Center for Cancer/Cancer Hospital Chinese Academy of Medical Sciences and Peking Union Medical College Beijing China; ^2^ Henan Office for Cancer Control and Research The Affiliated Cancer Hospital of Zhengzhou University Henan Cancer Hospital Zhengzhou China; ^3^ Department of Oncology Kailuan General Hospital Tangshan China; ^4^ Health Department of Kailuan (group) Kailuan General Hospital Tangshan China; ^5^ Department of Thoracic Surgery National Cancer Center/National Clinical Research Center for Cancer/Cancer Hospital Chinese Academy of Medical Sciences and Peking Union Medical College Beijing China

**Keywords:** China, colorectal neoplasms, risk prediction, risk score

## Abstract

To build a simple predictive model as a guide to stratify average‐risk population for colonoscopy examinations. We collected data from 92 923 males without a prior history of cancer enrolled in the Kailuan Cohort Study of China. Risk factors included in the evaluation of colorectal cancer (CRC) were collected by questionnaire‐based interviews at the baseline. Logistic regression coefficients for incident CRC predictors were converted into risk scores by the absolute value of the smallest coefficient in the model and rounding up to the nearest integer. Receiver operating characteristic (ROC) analysis with the leave‐one‐out cross‐validation method was applied to evaluate model performance. In the 10‐year follow‐up, 353 CRC patients were in the cohort. Age, alcohol consumption, waist circumference, occupational sitting time, and history of diabetes were selected for the scoring system, and the adjusted area under the ROC was 0.66. Population in the highest risk group (16‐19 points) had a 33.12‐fold (95% CI: 13.44‐81.59) higher risk of CRC than those in the lowest risk group. When we defined 13 points as the cut‐off, the sensitivity and specificity of the scoring system for CRC were 67.99% and 62.42%, respectively. A simple scoring system for CRC has been developed to identify men at an increased relative risk of CRC within 10 years using several well‐established risk factors, which allows selection of asymptomatic candidates for priority of CRC screening and saving the health resource in cancer prevention and control.

## Graphical abstract

In a cohort study, the authors developed a simple scoring system for colorectal cancer that identifies men at a relatively increased risk of colorectal cancer within 10 years on the basis of several well‐established risk factors (age, alcohol consumption, waist circumference, occupational sitting time and history of diabetes). Those in the highest risk group (16‐19 points) had a 33.12‐fold higher risk of colorectal cancer than subjects in the lowest risk group.

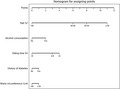

## 1 INTRODUCTION

Colorectal cancer (CRC) is the fourth most common cancer in men and the third most common cancer in women in China,^1^ with an age‐standardized incidence rate of 18.02 per 100 000 in 2015; additionally, CRC is the fifth most common cause of death from cancer with an age‐standardized mortality rate of 8.21 per 100 000. In recent decades, the incidence and mortality rates of CRC have shown an increasing trend in China, which could be explained by changes in diet, adoption of a westernized lifestyle and deficiency in early detection and early treatment of cancer.^2^, ^3^ Randomized controlled trials (RCT) and observation studies have shown that endoscopic or stool‐based screening carries considerable potential for reducing the burden of this disease.^4^, ^5^, ^6^ Unfortunately, population‐based endoscopy screening programs are unlikely to be cost‐effective or achievable due to the low incidence of CRC and the cost, invasiveness, and low screening rate.^7^ Thus, methods to identify high‐risk population for CRC and increase adherence to screening recommendations are desperately needed.

Risk assessment tools are generally recommended for selecting high‐risk patients for colonoscopy based on well‐established biological or behavioural factors. Tools for breast and prostate cancers have been helpful in making clinical decisions for screening and prevention strategies.^8^, ^9^ However, for CRC cancer, though numerous prediction models have been established, almost no prediction tools specific to the Chinese population have been developed based on lifestyle and risk factor exposure in mainland China.

Thus, aiming to develop a risk score with simple factors for CRC in China, we seek to develop a practical tool for CRC screening based on a large prospective cohort of Chinese men with 10‐year follow‐up period.

## 2 MATERIALS AND METHODS

### 2.1 Ethical statement

This study was jointly approved by the ethics committee of Cancer Hospital, Chinese Academy of Medical Sciences (CHCAMS) and that of Kailuan General Hospital. Kailuan General Hospital was responsible for the ethical approval of research conducted not only at its site but also at 10 other hospitals, which were also affiliated with the Kailuan Group; in addition, Kailuan General Hospital was in charge of the routine patient check‐up and the cohort study. Written informed consent was obtained from all the subjects at their baseline interview visit.

### 2.2 Study design and population

Our data were obtained from the Kailuan Cohort Study of China, which is a health examination of employees of the Kailuan Company in Tangshan City in Hebei Province, about 90 miles southeast of Beijing. In the past few decades, Kailuan Group has developed a comprehensive company that manages coal production, machine manufacturing, transportation, chemical production, education, and healthcare, etc The methodology for the Kailuan study have been described previously.^10^, ^11^, ^12^ In short, since May 2006, more than 13 000 people over the age of 18 have participated in biennial questionnaire interviews and clinical examinations at 11 hospitals affiliated with Kailuan Company.

Participants who met the criteria as follows were included in this cohort study: (a) signed informed consent, (b) were males over the age of 18 years, (c) completed the interview with the questionnaire and provided basic information on demographics and lifestyle factors. In cases of potential survival bias, subjects diagnosed with cancer prior to the initial interview were excluded from the study. Finally, a total of 92,923 men were included in this study.

### 2.3 Definitions of potential predictors

A face‐to‐face interview was performed at each visit by a trained physician or nurse with a standardized questionnaire, which included information on demography and possible risk factors associated with CRC. The baseline information obtained from the first interview was used in the final analysis. Smoking was defined as smoking at least 1 cigarette per day for more than 6 months. Alcohol consumption was defined as drinking at least once a month for more than 6 months. Body weight and height were measured while subjects wore lightweight non‐footwear, and the Body Mass Index (BMI) was calculated as follows: BMI = weight (kg)/height (m^2^). The following self‐declared basic variables were included in the analysis: categorical age in years (<40, 40‐49, 50‐59, or ≥60); history of smoking (never, former, or current); BMI (<18.5 kg/m^2^, 18.5‐24.9 kg/m^2^, 25.0‐29.9 kg/m^2^, or ≥30.0 kg/m^2^); alcohol consumption (yes or no); waist circumference (<95 cm or ≥95 cm); occupational sitting time (<4 h/d, 4‐8 h/d, or >8 h/d); and diabetes’ diagnosis history (yes or no).

### 2.4 Determination of cases of colon and rectal cancer

The study end point was the development of colon or rectal cancer within the 10‐year follow‐up period. During the study period, by tracking subjects when they participated in routine medical examinations every two years, incident colon or rectal cancer cases were collected until December 31, 2015. Besides, incident cases of CRC were identified by linking records with the Tangshan Health Insurance System, death cases were identified by linking records with the Kailuan Social Security System and discharge summaries from the eleven affiliated hospitals where participants were diagnosed and treated each year to obtain any outcome information that could have been missed. The diagnosis of incident CRC was confirmed by a review of medical records and pathology reports by clinical experts. Cancer was coded according to the International Classification of Diseases, tenth revision (ICD‐10); colon cancer was coded as C18 and rectal cancer was coded as C19‐C20.

### 2.5 Statistical analyses

Exact person‐years (PYs) of follow‐up were calculated from the date of baseline interview to the date of incidence, death or termination of follow‐up (31 December 2015). The world standard population used in our study was that as proposed by Segi.^13^

Descriptive analysis, expressed as proportions for categorical variables, was conducted to compare the baseline characteristics of those with and without the CRC occurrence. For categorical variables, Chi‐squared tests were used to determine the univariate association between the baseline factors and CRC development. The data management and all analyses were performed using SAS statistical software, version 9.4 (SAS Institute Inc, Cary, NC).

Multivariable logistic regression was used with backward stepwise selection of *P* < .20 for entry of variables and *P* > .20 for removal of variables to identify CRC independent predictors. Risk scores were created by dividing beta coefficients by the absolute value of the smallest coefficient in the model and rounding up to the nearest integer to generate a simple integer‐based point score for each predictor variable. For each participant, the total score was calculated by adding each component together. The cohort was divided into risk categories that corresponded to each risk score.

Discrimination of the model, risk scores, and risk categories was assessed using an analysis of receiver operating characteristic (ROC) with the leave‐one‐out cross‐validation (LOOCV) method,^14^ in which N‐1 samples (of the N samples in the data) were trained to predict the probability of the Nth remaining sample being CRC. The Hosmer‐Lemeshow goodness‐of‐fit statistic was used to evaluate the reliability of the models,^15^ where a *P* > .05 indicated adequate calibration. We examined the calibration of risk categories by calculating the number of observed events relative to the expected number in each risk class. Based on the final predictor model, the predicted probability of the outcome for each member of the cohort was calculated. These risks were summed to produce an average predicted risk within each risk category, and was compared with the observed incidence of the outcome in the same risk class. For individuals above each point‐based cut‐off threshold, the adjusted area under the ROC (AUROC), the sensitivity, the specificity, the Youden's index (sensitivity + specificity −1), the risk of CRC within 10 years (equivalent to positive predictive value), and the number of referrals for additional screens per cancer correctly predicted were assessed.

## 3 RESULTS

### 3.1 Baseline characteristics of participants

During the 10‐year follow‐up period, a total of 353 of the 92 923 males in the Kailuan Cohort Study developed CRC. The CRC incidence rate was 47.49/100 000 PYs in males, with an age‐standardized rate (ASR) of 20.19/100 000 PYs. Those who developed CRC were significantly older (mean age 59 vs 52 years), and more likely to consume alcohol and have abdominal obesity (waist circumference ≥ 95 cm), a lower occupational sitting time, and a history of diabetes. However, in univariate analyses, the history of smoking and BMI were not significant predictors. The basic characteristics of participants and the univariate analyses results among groups are presented in Table 1.

**Table 1:** Baseline characteristics of 92 923 men with 10 y of follow‐up and incident cases of colorectal cancer

Characteristics	Total, n (%)	No colorectal cancer, n (%)	Colorectal cancer, n (%)	*χ* ^2^	*P‐*value
Age (y)				161.76	<.001
<40	17 124 (18.43)	17 119 (18.49)	5 (1.42)		
40‐49	21 181 (22.79)	21 137 (22.83)	44 (12.46)		
50‐59	32 866 (35.37)	32 730 (35.36)	136 (38.53)		
≥60	21 752 (23.41)	21 584 (23.32)	168 (47.59)		
History of smoking				1.80	.406
Never	52 116 (56.09)	51 918 (56.09)	198 (56.09)		
Former	3964 (4.27)	3944 (4.26)	20 (5.67)		
Current	36 843 (39.65)	36 708 (39.65)	135 (38.24)		
Alcohol consumption				20.48	<.001
No	73 341 (78.93)	73 097 (78.96)	244 (69.12)		
Yes	19 582 (21.07)	19 473 (21.04)	109 (30.88)		
BMI (kg/m^2^)				0.39	.942
<18.5	2274 (2.45)	2267 (2.45)	7 (1.98)		
18.5‐24.9	54 711 (58.88)	54 503 (58.88)	208 (58.92)		
25.0‐29.9	31 760 (34.18)	31 639 (34.18)	121 (34.28)		
≥30.0	4178 (4.50)	4161 (4.49)	17 (4.82)		
Waist circumference (cm)				18.99	<.001
<95	71 912 (77.39)	71 673 (77.43)	239 (67.71)		
≥95	21 011 (22.61)	20 897 (22.57)	114 (32.29)		
Occupational sitting time (h/d)				5.84	.054
<4	67 606 (72.75)	67 337 (72.74)	269 (76.20)		
4‐8	22 258 (23.95)	22 178 (23.96)	80 (22.66)		
>8	3059 (3.29)	3055 (3.30)	4 (1.13)		
Diabetes				4.57	.033
No	90 021 (96.88)	89 686 (96.88)	335 (94.90)		
Yes	2902 (3.12)	2884 (3.12)	18 (5.10)		

### 3.2 CRC risk prediction model

**Figure 1:**
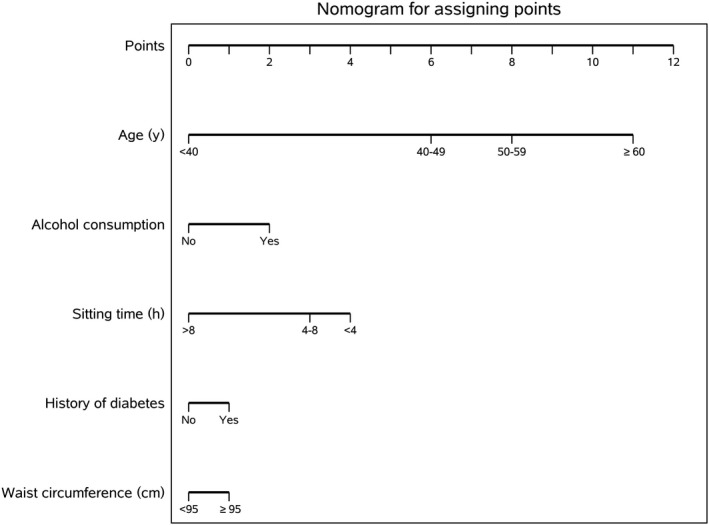
Nomogram for assigning points (out of a total of 19) to help identify individuals at a higher risk for colorectal cancer

Independent predictors for the final multivariable model, after applying the multi‐phase stepwise procedure to predict CRC development within 10 years, were age at baseline, drinking status, waist circumference, occupational sitting time, and history of diabetes. The adjusted predictors of CRC are displayed in Table 2. The efficacy of the multivariable model was compared with that obtained by a sum of risk factor. The score was assigned to each risk factor for CRC as follows (Figure 1): age < 40 years (0), 40‐49 years (6), 50‐59 years (8), and ≥ 60 years (11); non‐drinking (0) and drinking (2); waist circumference < 95 cm (0) and ≥ 95 cm (1); occupational sitting time > 8 h/d (0), 4‐8 h/d (3), and < 4 h/d (4); and no history of diabetes (0) and history of diabetes (1). Each risk factor has been allocated by one score, then the sum of all risk factors for ranged from 0 to 19 per participant. With an unadjusted AUROC of 0.71 (95% CI, 0.69‐0.73) and an adjusted AUROC of 0.66 (95% CI, 0.63‐0.68) for the LOOCV method, the model showed good discrimination. The results for goodness‐of‐fit test (Hosmer‐Lemeshow test, *χ* ^2^ statistic = 6.12, *P* = .634) demonstrated good performance of the model.

**Table 2:** Multivariable predictors of colorectal cancer

Predictor	Beta coefficient	OR (95% CI)	Prediction score points
Age (y)			
<40	—	1.00	0
40‐49	1.50	4.47 (1.76‐11.37)	6
50‐59	2.16	8.64 (3.52‐21.19)	8
≥60	2.80	16.41 (6.71‐40.13)	11
Alcohol consumption			
No	—	1.00	0
Yes	0.54	1.72 (1.34‐2.21)	2
Waist circumference (cm)			
<95	—	1.00	0
≥95	0.27	1.30 (1.02‐1.67)	1
Occupational sitting time (h/d)			
>8	—	1.00	0
4‐8	0.92	2.52 (0.79‐8.04)	3
<4	1.10	2.99 (0.96‐9.37)	4
Diabetes			
No	—	1.00	0
Yes	0.36	1.43 (0.87‐2.35)	1

Abbreviations: 95% CI, 95% confidence interval; OR, odds ratio.

A risk class model was built to identify those at lowest (0‐5 points), low (6‐10 points), high (11‐15 points), and highest (16‐19 points) risk of CRC. There was an exponential trend of increased risk of CRC along with increasing points. Compared with those in the lowest risk group (0‐5 risk points), those in the highest risk group (16‐19 points) had an odds ratio (OR) of 33.12 (13.44‐81.59) for CRC. The observed and predicted CRC rates were in close agreement across the 4 categories. The observed risk of CRC over 10 years was 0.03% in the lowest risk group, 0.12% in the low‐risk group, 0.45% in the high‐risk group, and 1.04% in the highest risk group. The detailed description and performance of the risk classes could be found in Table 3.

**Table 3:** Performance of 4 risk groups for colorectal cancer

Risk group	Total, n (%)	Predicted CRC, n	Observed CRC, n	Predicted OR (95% CI) of CRC	Observed 10‐y risk of CRC (%) (95% CI)
0‐5 points	15 713 (16.91)	6	5	1.00	0.03 (0.00‐0.06)
6‐10 points	15 465 (16.64)	26	19	3.86 (1.44‐10.35)	0.12 (0.07‐0.18)
11‐15 points	53 408 (57.48)	234	242	14.30 (5.90‐34.66)	0.45 (0.40‐0.51)
16‐19 points	8337 (8.97)	87	87	33.12 (13.44‐81.59)	1.04 (0.83‐1.26)

Abbreviations: 95% CI, 95% confidence interval; OR, odds ratio.

The discriminative performances at each point‐based cut‐off threshold are provided in Table 4. With the highest Youden's index of 0.30, a cut‐off threshold of 13+ points had a sensitivity of 67.99%, a specificity of 62.42%, and a positive predictive value of 0.69% and correlated with 146 referrals for further screening for every CRC predicted, with 37.70% (35,030 individuals) of the cohort deemed at a high risk.

**Table 4:** Statistics for performance of a points‐based colorectal cancer risk‐prediction model at different points‐based cut‐off values

Points cut‐off	Patients deemed high risk, n (%)	True cancers predicted (out of 353)	Sensitivity	Specificity	Youden's index	Absolute 10‐y risk of CRC per 100,000	Referrals for additional screens per CRC predicted	OR (above vs below cut‐off) (95% CI)
6+	77 210 (83.09)	348	98.58	16.97	0.16	450.72	221.87	14.22 (5.88‐34.38)
7+	75 648 (81.41)	348	98.58	18.66	0.17	460.03	217.38	15.95 (6.60‐38.57)
8+	75 315 (81.05)	348	98.58	19.02	0.18	462.06	216.42	16.33 (6.76‐39.48)
9+	74 733 (80.42)	348	98.58	19.64	0.18	465.66	214.75	17.00 (7.03‐41.10)
10+	72 116 (77.61)	343	97.17	22.47	0.20	475.62	210.25	9.94 (5.30‐18.64)
11+	61 745 (66.45)	329	93.20	33.65	0.27	532.84	187.67	6.95 (4.59‐10.52)
12+	53 882 (57.99)	303	85.84	42.12	0.28	562.34	177.83	4.41 (3.27‐5.95)
13+	35 030 (37.70)	240	67.99	62.42	0.30	685.13	145.96	3.53 (2.82‐4.41)
14+	27 251 (29.33)	202	57.22	70.78	0.28	741.26	134.91	3.24 (2.62‐4.00)
15+	20 000 (21.52)	163	46.18	78.57	0.25	815.00	122.70	3.15 (2.55‐3.88)
16+	8337 (8.97)	87	24.65	91.09	0.16	1043.54	95.83	3.34 (2.62‐4.26)
17+	3118 (3.36)	37	10.48	96.67	0.07	1186.66	84.27	3.40 (2.42‐4.79)

Abbreviations: 95% CI, 95% confidence interval; OR, odds ratio.

## 4 DISCUSSION

Based on this large prospective cohort of Chinese males, a prediction model, using age, alcohol consumption status, waist circumference, occupational sitting time and history of diabetes, has been developed to predict the development of CRC over a 10‐year follow‐up period. According to the well calibrated model, compared with those in the lowest risk group, those in the highest risk group were 33 times more likely to develop CRC.

The risk factors identified in our model for CRC are consistent with the findings of other multivariable analyses in men,^16^, ^17^, ^18^, ^19^ women,^20^, ^21^, ^22^ and both sexes.^17^, ^18^, ^23^, ^24^ Regarding age‐related risk stratification, our study showed that older age was the main risk factor for CRC and that the risk was more than sixteen times higher in the age group of more than 60 years than in the age group of less than 40 years. This means that screening people with the right age for CRC screening is critical. In our cohort, alcohol consumption, waist circumference ≥ 95 cm and diabetes were independent risk factors for CRC, and they are close to those found in other large prospective studies of men.^17^, ^19^, ^25^ Occupational sitting time was identified to be related to CRC incidence, but the results remain controversial, especially for rectal cancer.^26^ For rectal cancer, most studies did not find a negative correlation with occupational sitting time,^23^, ^27^, ^28^ which was consistent with our findings.

The target population we studied had a lower incidence of CRC than the average incidence in the Chinese population^1^ and had its own characteristics. Previous models, such as the Asia‐Pacific Colorectal Screening (APCS) score model,^29^ Physician's Health Study (PHS) score model^16^ and Korean Colorectal Screening (KCS) score model,^30^ were based on common factors such as smoking status and BMI. However, as shown in Table 1, smoking and BMI distributions in our study are consistent across non‐CRC and CRC populations. Finding new risk factors and further defining the CRC risks of this kind of population based on these factors is the focus of our research. We have chosen widely accepted, independent predictors that are easy to describe. Once validated in other populations, our model might have good applications in stratifying populations into risk groups for differential screening strategies or improving the cost effectiveness. Despite the low cost of administration, the absolute risk of CRC over 10 years for individuals exceeding the threshold of 13 points (685.13 per 100,000) remains quite low, which could limit the cost‐effectiveness of orientation of these individuals towards further screening.

Specific limitations and strengths deserve careful attention when interpreting our results. A major limitation of our study was that we could not externally validate the prediction rule for CRC and decided against data splitting to have more power identifying potential risk factors. We internally validated the model and chose only biologically plausible risk factors, so we are confident that our prediction model is generalizable to other male populations. Second, we lacked information about family history of colon cancer or rectal cancer for first‐degree relatives in baseline. The inclusion of such information would increase the predictive power of our model, but we did not expect this information to alter the relative risk values ​​of the factors we chose to include. The strengths of our study are its prospective nature, its large number of events and participants as well as its long follow‐up period. All of these strengths have provided enough power to detect relatively modest effects and minimize the potential bias caused by a preclinical disease. Second, patients with CRC were confirmed with double checks by clinical experts. Third, comprehensive information on potential confounding factors, such as cigarette smoking, alcohol consumption, BMI, waist circumference and history of diabetes, was collected. Finally, to solve the problems related to overfitting, the LOOCV method was used to assess the performance of our prediction model.

In summary, we created an easy‐used risk scoring system to predict 10‐year CRC risk in a large prospective cohort of Chinese men. Future research should validate this scoring system among other screening populations and evaluate the cost‐effectiveness of this approach.

## CONFLICTS OF INTEREST

All authors have no conflicts of interest to disclose.

## AUTHOR CONTRIBUTIONS

NL, MD, SW, and JH conceived and designed the study concept. GW, ZL, XF, YC, SC, JS, JR, CL, and XY carried out the acquisition and quality control of data. LW, XL, YW, and ML performed the statistical analysis or interpretation of data. LG performed the writing and drafting of the manuscript. HC, NL, and MD critically revised the manuscript for important intellectual content. All authors agreed to be accountable for the content of the work.

## DATA SHARING

The datasets for this manuscript are not publicly available because all our data are under regulation of the National Cancer Center of China. Requests to access the datasets should be directed to Min Dai, daimin2002@hotmail.com.
